# Benign and severe early-life seizures: a round in the first year of life

**DOI:** 10.1186/s13052-018-0491-z

**Published:** 2018-05-15

**Authors:** Piero Pavone, Giovanni Corsello, Martino Ruggieri, Silvia Marino, Simona Marino, Raffaele Falsaperla

**Affiliations:** 1 Department of Clinical and Experimental Medicine, Section of Pediatrics and Child Neuropsychiatry, A.U.O. Vittorio Emanuele–Policlinico of Catania, Via Santa Sofia 78, 95100 Catania, Italy; 2 Department of Maternal and Child Health, University of Palermo, Palermo, Italy; 3 University-Hospital ‘Policlinico-Vittorio Emanuele, University of Catania, Catania, Italy

**Keywords:** Infantile epilepsy, Epileptic encephalopathies, Early onset seizures, Seizures

## Abstract

At the onset, differentiation between abnormal non-epileptic movements, and epileptic seizures presenting in early life is difficult as is clinical diagnosis and prognostic evaluation of the various seizure disorders presenting at this age. Seizures starting in the first year of life including the neonatal period might have a favorable course, such as in infants presenting with benign familial neonatal epilepsy, febrile seizures simplex or acute symptomatic seizures. However, in some cases, the onset of seizures at birth or in the first months of life have a dramatic evolution with severe cerebral impairment. Seizure disorders starting in early life include the “epileptic encephalopathies”, a group of conditions characterized by drug resistant seizures, delayed developmental skills, and intellective disability. This group of disorders includes early infantile epileptic encephalopathy also known as Ohtahara syndrome, early myoclonic encephalopathy, epilepsy of infancy with migrating focal seizures, infantile spasms syndrome (also known as West syndrome), severe myoclonic epilepsy in infancy (also known as Dravet syndrome) and, myoclonic encephalopathies in non-progressive disorder.

Here we report on seizures manifesting in the first year of life including the neonatal period. Conditions with a benign course, and those with severe evolution are presented. At this early age, clinical identification of seizures, distinction of each of these disorders, type of treatment and prognosis is particularly challenging.

The aim of this report is to present the clinical manifestations of each of these disorders and provide an updated review of the conditions associated with seizures in the first year of life.

## Background

Seizures are not uncommon clinical manifestations in childhood and a frequent reason for consultation in child neurology. In infancy, movements during epileptic seizures are often subtle, unnoticed by parents and, not easily recognized. Seizures are caused by abnormal and excessive discharges of neurons, usually self-limited, and often accompanied by abnormal behavior, and sensory-motor manifestations [1]. The term epilepsy defines the recurrences of two or more unprovoked seizures [2]. Seizure types have been recently classified according to their site of onset in focal, generalized and unknown. The focal seizures are further distinguished in: aware or with impaired awareness, with motor or non-motor onset, and focal to bilateral tonic-clonic; the generalized, in motor tonic-clonic, other motor and non-motor (Absence); the unknown in motor tonic-clonic, other motor and non-motor, and unclassified. [3, 4]. Various etiological events cause seizures in childhood, the most common being fever, infections, head injury, metabolic dysregulation, noxious perinatal events (e.g., stroke), and hypoxic-ischemic-encephalopathies [5]. Less frequent causes of seizures in childhood include chromosomal deletions, and duplications, cerebral malformations by selected single gene mutations and inborn errors of metabolism [2]. The prenatal and early life periods are critical for brain development with rapid involving synaptogenesis, dendritic arborization, myelination, apoptosis, and priming of excess process, and synapses, all factors which concur for the normal cerebral growth [6]. Any pathologic events during this early period of life might result in severe cerebral impairment with developmental delay and intellectual disability, often associated with epileptic seizures and various comorbidities [7, 8]. Seizures occurring in the first year of life might have an evolution ranging from benign to severe [9, 10]. Typically benign cases of seizures occurring in the first year of life include Benign Familial Neonatal Epilepsy (BFNE), Febrile Seizures simplex (FSs) and Acute Symptomatic Seizures (ASS). Febrile Seizure complex (FSc) might have a variable prognosis not always predictable. Severe seizures presenting at an early age include the epileptic encephalopathies a group of disorders defined on the basis of “the notion that epileptic activity may contribute to severe neurocognitive and behavioral dysfunction above and beyond what would be expected from the underlying pathology alone”. These impairments may have a progressive course over time [11, 12]. Clinical identification of the seizures by the parents and caregivers is challenging, as are diagnosis and prognostic counseling by the pediatricians and specialists in this field. This report aims to present the clinical manifestation of each of these disorders and provide an updated review of the conditions associated with seizures in the first year of life.

## Early life seizure with usually benign course

### Benign familial neonatal epilepsy

Unlike most cases of neonatal seizures, which have a high frequency of disability, the neonatal prognosis is usually benign in families affected by Benign Familial Neonatal Epilepsy (BFNE) [10, 13]. The clinical features of this disorder are relatively typical: the seizures begin in the first days of life in otherwise healthy looking babies and are typically associated with a family history of neonatal seizures. The affected infants tend to have a normal course in the developmental stages and the seizures tend to gradually disappear within the first months of life. [13–15]. The condition is inherited as an autosomal dominant trait with both genders being affected, and has been associated with a mutation in the KCN gene, localized on chromosome 20q13.33 [16]. With the term “KCNQ2 related epilepsy” are designed clinical conditions encompassing the classical type of BFNE, the Benign Familial Neonatal Infantile Seizures (BFNIS) and the Benign Familial Infantile Seizures (BFIS) [17, 18]. KCNQ2 related disorders are also reported in patients with epileptic encephalopathies [19]. Recently, disorders caused by BFNE have also been reported in individuals with KCNQ3 gene mutations [20]. A large family has been followed for three generations by some of the authors of the present article. In this family, not all of the members showed the classic evolution of the disorder: in one the seizures started at the age of 3 months, another member showed febrile seizures complex with EEG anomalies, and one suffered from focal seizures lasting until the age of 10 years [21].

### Acute symptomatic seizures

Seizures might be provoked by factors with onset in close temporal relationship with a well-documented brain insult. These events are referred to Acute Symptomatic Seizures (ASS) [22], as Situation-Related-Seizures (SRS) or also named as occasional, or reactive, or provoked seizures. The 1989 classification of the ILAE includes these events in the category of situation-related seizures because they show an identifiable proximate cause and do not typically recur spontaneously [23]. Aside from the febrile seizures and the infections directly involving the central nervous system, the seizures might manifest following trauma, intoxication, or anomalous administration of drugs. Other factors inducing ASS include electrolytic dysregulation, acute hypoglycemia, hypocalcemia, and hyponatremia. The occurrence of the ASS is particularly high in the infantile period since, at this age the brain seems to be more susceptible to such insults. The seizures present most frequently as motor tonic-clonic generalized types. Focal or unilateral types are uncommon. Recently, children with manifestations of transient generalized seizures have been reported in association with wild gastroenteritis [24]. A new pathogenetic pathway, the so-called “gut-brain axis”, has been reported as a causative event of seizures. Falsaperla et al. [25] have reported a 10-month-old male infant with seizures secondary to cow’s milk protein allergy. Neurologic signs disappeared after the suspension of the cow’s milk protein.

### Febrile seizures simplex

Febrile Seizures (FS) are the most common convulsive manifestations in childhood, affecting 4–6% of the pediatric population [26]. FS are classified as simplex or complex [27].

FSs are defined as a short (< 15 min.) generalized seizures, not recurring within 24 h which occur during a febrile illness not resulting from an acute disease of the nervous system, in a child aged between 6 months and 5 years, with no neurologic deficits and no previous afebrile seizures [27, 28]. Differential diagnosis is made with viral meningitis in the presence of positive neurologic signs, persisting loss of consciousness, and post-ictal drowsiness. In one/third of cases and until the age of 5 years, the seizures tend to reappear with other episodes of fevers. The evolution in epileptic seizures is rare, and almost similar to that of the general population, and no persistent residual signs of motor, behavioral, and cognitive disturbances are reported. In FSs, EEG recordings and brain MRI are not necessary, while the lumbar puncture is advised to be performed in FSs children less than 1-year-old and those under antibiotic treatment. Intravenous, intramuscular, buccal, intranasal or rectal benzodiazepines are administered to stop the crises [28–30]. Prophylactic pharmacologic treatment is not advised [28].

## Early life seizure with prognosis not predictable

### Febrile seizures complex

Children with Febrile Seizures complex (FSc) have characteristic clinical features opposite to those reported in FSs. The principal features of the manifestations are focal, or generalized, and prolonged seizures lasting more than 15 min, recurrence can happen within 24 h in the course of the same febrile episode, the temperature might not be elevated. Moreover the crises might be associated with post-ictal neurologic abnormalities, most frequently post-ictal palsy, or manifest in subjects with previous neurologic deficits [28, 29]. FSc might manifest in different ways, with onset before the first year of life or after 5 years, it might be present in alternation with afebrile seizures, or in members of a family affected by Genetic Epilepsy with Febrile Seizures plus (GEFS+). Febrile status epilepticus might also be recorded [27–29]. Patients with brain damage are more affected than those without. Differential diagnosis is posed with cerebral abscesses, meningoencephalitis, cerebral vascular malformations, cortical thrombophlebitis, and autoimmune encephalitis. Diagnostic investigations should include routinary analysis, an EEG recording, lumbar puncture, and plasma electrolytes. Brain MRI might also be indicated in patients with focal seizures, and those with episodes that happened after 5 years of age. Brain MRI is advised in an emergency in patients who present focal post-ictal deficit and persisting loss of consciousness, and also in patients with immunodeficiency, or with seizures of particular long durations. The acute treatment is based on the use of benzodiazepines [30]. In FSc, prophylactic treatment might be useful in reducing the frequency and the duration of the crises but is not considered able to prevent the onset of subsequent epileptic seizures [29]. Very prolonged FSc is thought to be associated with mesial temporal sclerosis and temporal lobe epilepsy, but the direct relationship among these disorders and FSc remains uncertain [31, 32]. FSc might affect child members of a family affected by (GEFS+), a complex autosomal dominant disorder in which individuals present with genetic mutations of SCN2A (a voltage-gated sodium channel), or less frequently of SCN1B [33, 34]. With this mutation, individuals suffering from FSc might belong to a family in whom other members are affected by a variety of seizure types, such as myoclonic, tonic, and tonic-clonic seizures. One/third of the GEFS+ patients are reported to have febrile seizures, which manifest with crises of prolonged durations, presenting in children less than 2 years old and, with residual signs including post-ictal hemiplegia [33].

## Early life seizures with usually severe course

### Neonatal seizures

**Fig. 1:**
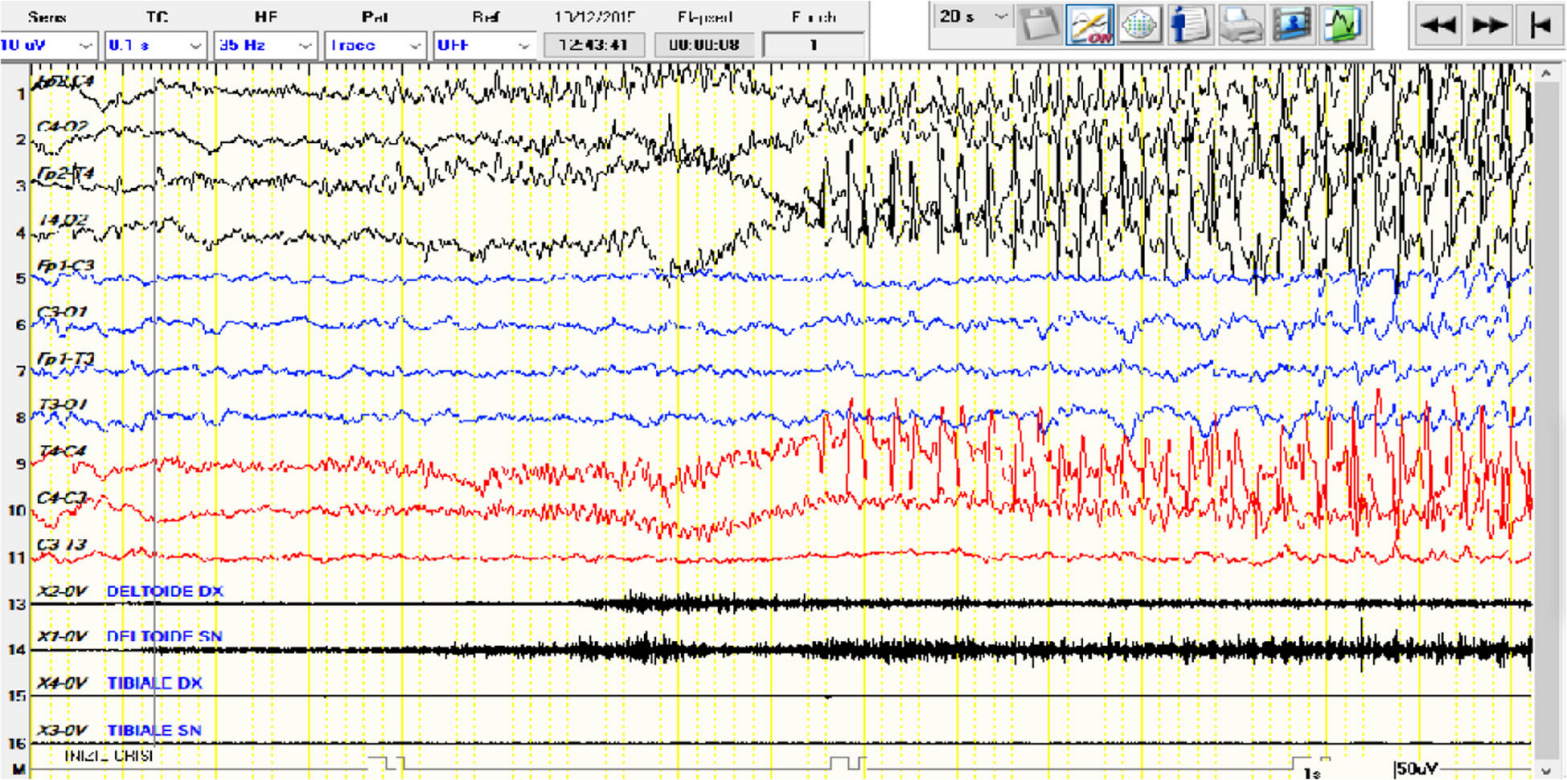
Four day old boy affected by stroke with neonatal seizures onset. The picture shows the focal discharges located on the right hemisphere

Neonatal seizures have a fairly high incidence, usually more than in any other period of life. In most cases the neonatal seizures are due to acute dysfunction of the cerebral nervous system and the most common cause is hypoxic-ischemic encephalopathies [35], followed by intracranial hemorrhage, infections and strokes. The incidence rate of neonatal seizures is 1–2% of life births [36, 37]. Neonatal seizures are distinguished according to the presentation as clinical seizures, electroclinical seizures and electroencephalographic seizures and based on the pathophysiology in epileptic and non-epileptic seizures [14]. EEG is particularly useful for diagnostic evaluation (Fig. 1).

Neonatal epilepsy syndromes are uncommon, and represent a sizable subgroup of neonatal seizure etiologies [38]. In this subgroup a genetic, metabolic, or structural cause might be identified, whereas in some cases the etiologic event cannot be determined using the current diagnostic investigation. The genetic causes of neonatal epilepsy are distinguished in: malformations of cortical development; genetic-metabolic; genetic vascular; genetic syndromic and genetic-cellular [38]. Neonatal seizures are also included in the cohort of neurodegenerative disorders, and in association with malformative cerebral syndromes. A study on a prospective cohort of newborns with seizures was conducted by Shellhaas et al. [39] on 611 patients enrolled in the Neonatal Seizure Registry US. The study includes neonates with seizures related to epileptic encephalopathies (without structural brain abnormalities), brain malformations, and benign familial epilepsies. Among the group enrolled, 79 (13%) had epilepsy, (35 epileptic encephalopathy, 32 congenital brain malformation, 11 benign familial neonatal epilepsy and 1 benign neonatal seizures). In this study, KCN2 variants were the most common genetic anomaly reported within the group of patients with epileptic encephalopathies.

The neonatal seizures may recognized an Inborn Error of Metabolism (IEM), as a putative cause. Among these the Pyridoxine-dependent epilepsy, the pyridox (am)ine 5′-phosphate oxidase (PNPO) deficiency, GLUT-1 (glucose transporter 1) deficiency, non-ketotic hyperglycinemia, maple syrup urine disease are the most quoted examples [40]. Pyridoxine-dependent epilepsy (PDE) is an autosomal recessive enzyme defect in the vitamin B6 metabolism. In this disorder biochemical and genetic studies are available for a correct diagnosis including elevated urinary alpha-aminoadipic semialdehyde excretion and ALDH gene mutation. Treatment consists of the use of pyridoxine, which has been also proposed as an initial diagnostic approach in cases of infantile refractory epilepsy of unknown cause [41]. Pyridox (am)ine 5′-phospate oxidase (PNPO) deficiency is due to the enzyme defect which converts pyridoxine 5′-phosphate and pyridoxamine 5′- phosphate (PLP) into pyridoxal 5′-phosphate (PLP). Treatment with pyridox-5′ phosphate in the first days of life has shown good therapeutic response [42, 43]. GLUT 1 (glucose transporter 1) deficiency presents with respiratory distress, hypotonia, absence of neonatal reflexes and less frequently with arthrogryposis or joint laxity. Diagnosis is related to a mildly elevated cerebrospinal fluid (CSF) glycine levels, and normal or slightly elevated serum or plasma glycine levels. Survey of variants in SLC6A gene might be diagnostic [44]. Non ketotic hyperglycinemia (NKH) is caused by a deficiency in the glycine cleavage system presenting with severe hypotonia and crisis of apnea [45]. The diagnosis is based on the elevated glycine concentrations in CSF, in association with an increased CSF/plasma glycine ratio [46]. Maple syrup urine disease (MSUD) is caused by deficit of branched-chain alfa ketoacid dehydrogenase complex. Mutations in the BGKDHA, BCKDHB, and DBTI genes have been associated with this disorder [47].

Except for PDE, PLP and PNPO no treatment is known for the above mentioned IEM conditions.

Regardless of the etiology of the neonatal seizures, prognosis in most of the cases is severe. In fact the brain is more susceptible in the neonatal period and in infancy than in older children and this vulnerability is linked to a more express activity age-dependent of receptors for excitatory rather than inhibitory neurotransmission [48]. For a long time, neonatal seizures were treated with phenobarbital and phenytoin as the first-line drugs. Recently, phenobarbital and phenytoin treatment in neonatal seizures has been questioned because of doubtful seizure control and for the consequence of long term alterations in brain structures [49, 50]. As demonstrated in rat CAT neurons, treatment with phenobarbital disrupts GABergic synaptic maturation [51]. Pharmacokinetic data for new drugs in treatment of neonatal seizures is limited. Treatment with levetiracetam has been proposed to be effective in a recent study [52].

### Epileptic encephalopathies

Epileptic encephalopathies include severe epileptic disorders that share similar characteristics: onset in early life, persistent electroencephalographic abnormalities, drug-resistant seizures of various types, and cognitive involvement. The definition also includes the condition that seizure activity per se, above and beyond the effects of underlying causal factors, interferes with the developmental skills of the affected children [4, 11, 12]. There is agreement that uncontrolled seizures might provoke a negative effect on the cerebral functions in the affected patients. This is confirmed by the observation that children unresponsive to treatment have a progressive decline along the course of the disorder. At the same time, it is well-known that children with West syndrome show a better prognosis when seizures have an early diagnosis and receive a precocious treatment [53, 54]. The same results have been reported in drug-resistant epileptic children submitted to surgical treatment, with the demonstration of progressive improvement of the cognitive function after the intervention [55, 56]. Certainly, the persistence of the epileptic seizures and unresponsiveness to the treatment is consistent with the worsening of the cognitive decline in the affected children, but the underlying etiologic factors are of notable importance in contributing to the deleterious brain effect. Epileptic encephalopathies are predominantly symptomatic and sporadic, and new technologies have identified several genes involved in the etiology of these disorders [57]. The epileptic encephalopathy manifesting in the first months of life include Early Infantile Epileptic Encephalopathy (EIEE) (also known as Ohtahara syndrome), Early Myoclonic Encephalopathy (EME), Epilepsy of Infancy with Migrating Focal Seizures (EIMFS), Infantile Spams Syndrome (ISS) (also known as West syndrome), Severe Myoclonic Epilepsy in infancy or Dravet Syndrome (DS), and Myoclonic Encephalopathies in non-progressive disorder [5, 11, 12, 53, 54].

#### Early infantile epileptic encephalopathy - early myoclonic encephalopathy

**Fig. 2:**
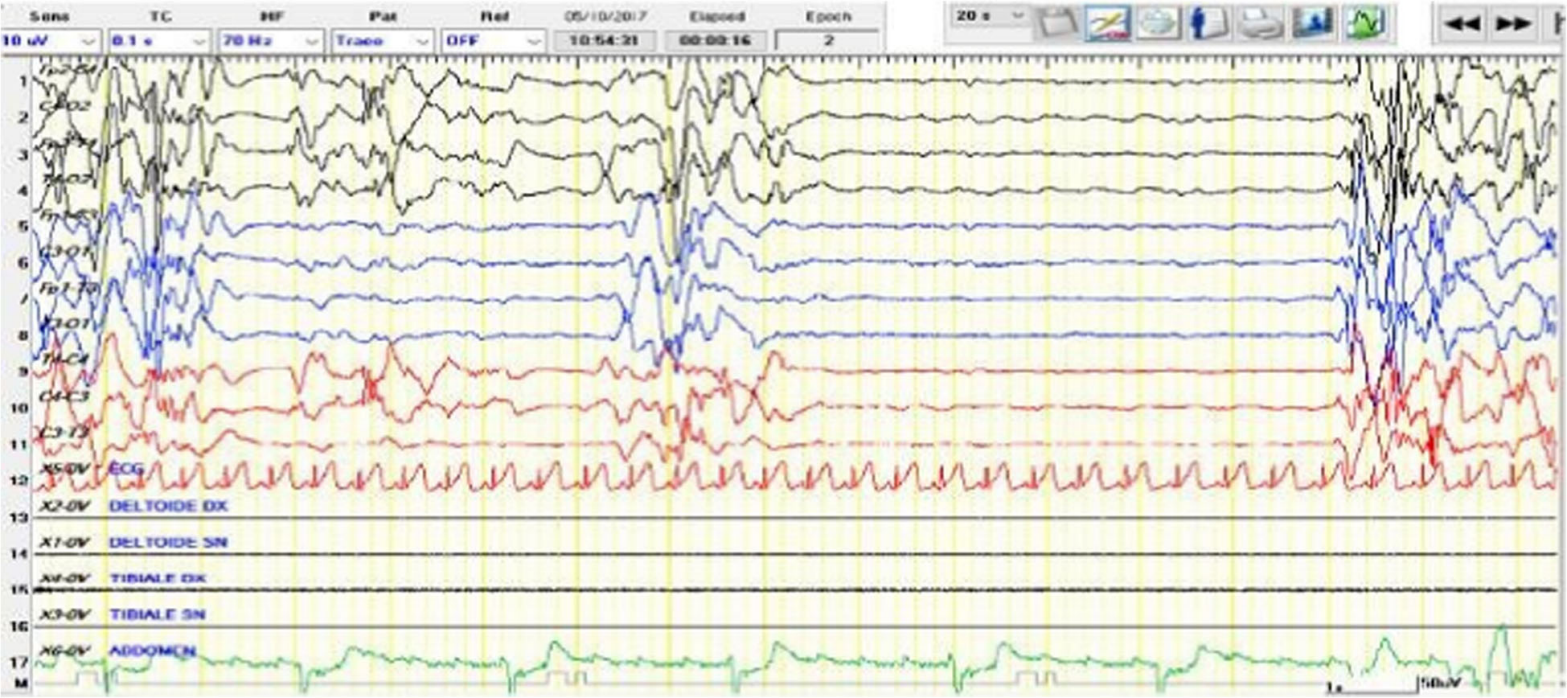
One month old female with Ohtahara syndrome: The EEG recording shows the typical burst-suppression pattern

Early Infantile Epileptic Encephalopathy was first reported by Ohtahara et al. in 1976 [58] and subsequently overviewed in 16 patients by Yamatogy and Ohtahara in 2002 [59]. The syndrome is clinically characterized by early onset seizures presenting in 30% of cases within the first 10 days of life, by tonic spasms as seizure types (either generalized and symmetrical or lateralized), and with less frequency by focal and myoclonic seizures. The intercritical EEG findings show high voltage bursts of slow waves mixed with multifocal spikes, with phases of flat suppression [59–61] (Fig. 2). Mutations in several genes have been implicated, including ARX, STXBP1, KCNQ2, SLC25A22, and CDKL5 [62–65]. Structural cerebral anomalies are often detected by brain MRI, including cerebral asymmetry, hemimegalencephaly, lissencephaly and focal-cortical dysplasia [61, 65, 66]. Hypotonia, severe developmental delay, and respiratory problems are associated with these seizure types. The prognosis is poor, with severe intellective delay, and resistance to drug treatment and to ketogenic diet. Transition to West syndrome is frequently observed.

The diagnostic criteria for Early Myoclonic Encephalopathy include early onset, and myoclonus as the main seizure type with frequent episodes of erratic partial seizures. Massive myoclonus, or tonic spasms have also been reported. The EEG shows a pattern of burst-suppression. Metabolic disorders are often recognized as a causative event of the disorder as well as brain malformations [67]. Mutation in SLC25A22, which encodes the mitochondrial glutamate/H+ symporter, has been associated with this encephalopathy [68]. Prognosis is poor. EIEE and EME share many common characteristics and the distinction between these epileptic disorders is questionable [69]. Covanis [70] claims that EME, contrarily to EIEE, might manifest with different characteristics: the tonic seizures are less frequent, the focal seizures (when present) tend to appear later, the myoclonic seizures are early onset, erratic, and frequently massives, and the EEG suppression is longer and the paroxysm shorter. The etiology is mainly of metabolic origin and less structural. Also, the transition of EME in West syndrome is not frequently reported.

#### Epilepsy of infancy with migrating focal seizures

The Epilepsy of Infancy with Migrating Focal Seizures (EIMFS) is also referred to as Malignant Migrating Partial Seizures in Infancy (MMPSI) [71, 72]. In most of the cases, EIMFS onset is reported in the firsts 6 months of life, with almost continuous migrating polymorphous focal seizures involving part of the body with hemi-lateralization and electrographic multifocal discharges. The prognosis is poor, with evolution to the other types of epileptic encephalopathies, such as Ohtahara syndrome and West syndrome. McTague et al. [73] reported the clinical features of 100 children affected by MMPSI. Among these children, focal motor seizures were reported at onset in 64%; focal seizures affecting alternating sides of the body in 59%; secondary generalization in 17%; and epileptic spasms in 7%. Moreover, autonomic features were also present in 43% and generalized tonic-clonic seizures in 8% of the cases. Hypotonia and reduction of the occipitofrontal circumference (OFC) and severe microcephaly was registered in most of the patients. Treatment with a high level of phenytoin gave fairly good results. Among the 14 patients reported by a National surveillance study in association with British Pediatric Neurology Surveillance Unit, at presentation, 10 infants had focal motor seizures mainly involving the face, eyes, and limbs with head-turning; the seizures were generalized in five, generalized tonic in three and clonic in two. An autonomic feature associated with seizures was reported in 12 out 14 cases. EEGs showed various patterns, ranging from subtle burst-suppression with rhythm decrement to hypsarrhythmia, according to the age of infant submitted to the EEG. In this group of patients, genetic analysis identified two patients with mutations in the newly discovered KCNT1 gene [73]. A study was conducted on 12 patients, and data from a further 34 collected from the literature by Howell et al. [74] with the aim of better defining the clinical aspect of early epileptic encephalopathies with SCN2A gene mutations. In the group of 12 patients, multifocal interictal epileptiform discharges were reported in all. At the onset of symptoms, seven patients showed EIMFS clinical expression and two of Ohtahara syndrome. The same authors [74] report in five patients, an improved seizure control with sodium channel blockers using high dosage of phenytoin. The authors [74] concluded that SCN2A encephalopathy is a frequent cause of EIMFS which might manifest with three clinical phenotypes: neonatal-infantile-onset groups (either with severe or intermediate outcomes) and childhood-onset. SCN2A is recognized by these authors as the second most common cause of EIMFS. Pharmacological treatment attempts have been carried out with several, known anticonvulsant drugs including vigabatrin, stiripentol, valproic acid and clobazam, but with poor results [74].

#### Infantile spasms syndrome

**Fig. 3:**
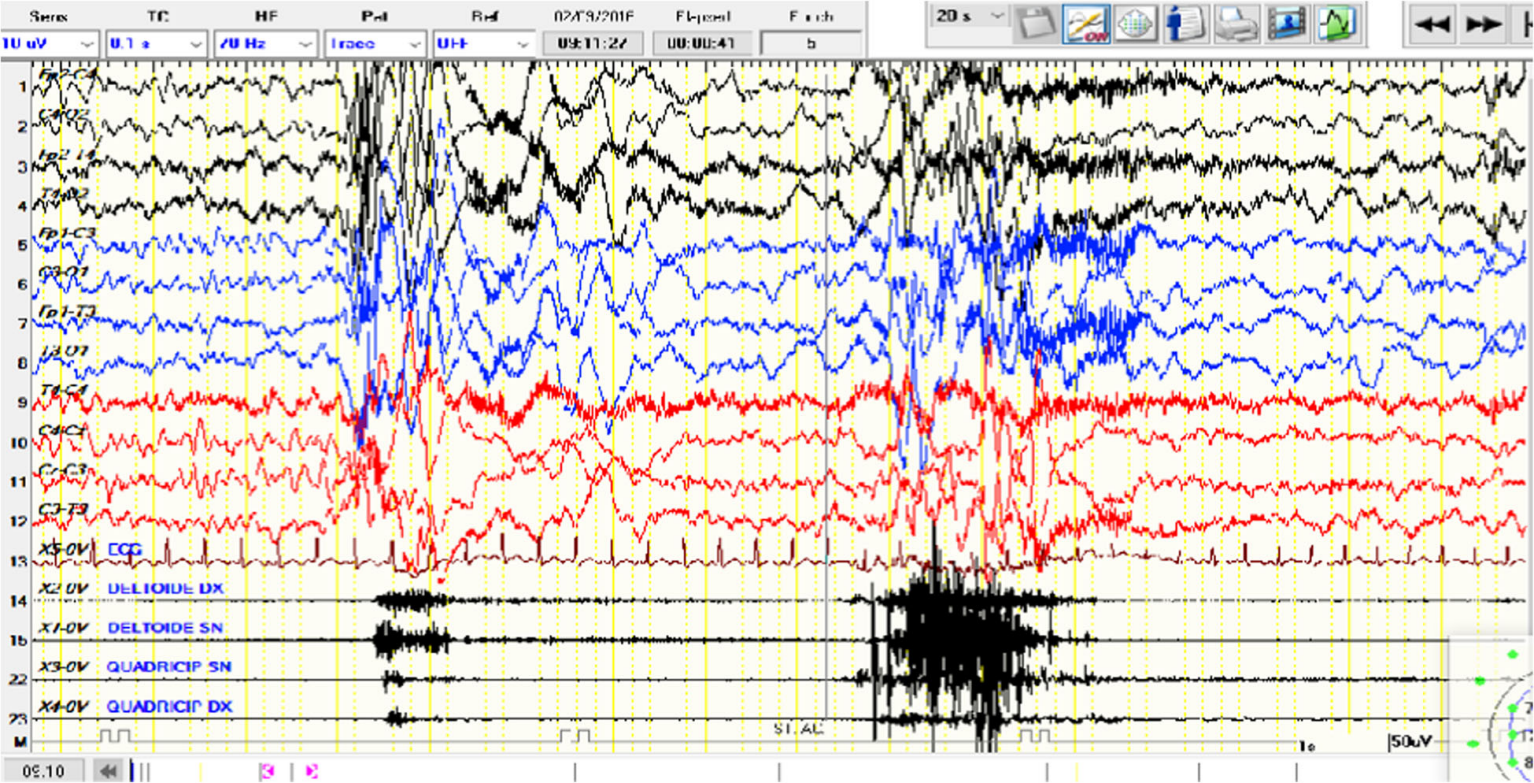
Six months female with ISS. The critical EEG shows the presence of synchronous and symmetric spike- wave discharges

West syndrome is defined by the classic triad of infantile spasms, hypsarrhythmia, and developmental arrest or regression. West syndrome is also indicated as “infantile spasms” and “epileptic spasms” because the spasms are the most notable event. However, recently this disorder has been indicated with the term “infantile spasms syndrome” (ISS). In fact, this term includes the onset of seizures largely prevailing in infancy, the seizure types, and the EEG findings as present features. As these features tend to occur together, and at the same time the application of the term “syndrome” seems to be correct [3, 75–77]. West syndrome, according to the new nomenclature, is considered a subtype of ISS since not all of the triad is always present at the same time. ISS is the most common among the group of epileptic encephalopathies. The estimated incidence of ISS is 2–3.5 per 10,000 live births [78]. In the typical manifestation, the seizures appear within the first year of life, usually between 4 and 6 months, with episodes of axial spasms of short duration occurring in clusters and at awakening. Psychomotor delay might precede, follow or coincide with the spasms. In rare occasions, the spasms might not manifest clearly and might express with less obvious signs, which are referred to as “subtle spasms”. The intercritical EEG presents with a high voltage arrhythmia and asynchronous, slow, and sharp waves, in a chaotic distribution with multifocal spikes and poly-spikes. Critical EEG might show a pattern of synchronous and symmetric spike- wave discharges (Fig. 3). Atypical modified hypsarrhythmia might be observed at the intercritical EEG with a pattern of asymmetric features, focal discharges, and semi-periodic burst- suppression [75]. Prognosis in most of the cases is severe, both for the control of seizures and for intellective delay. The underlying causes of ISS are numerous. Symptomatic causes are the most common, being identified in about 60–70% of cases. Among these, the most frequent are the outcomes of hypoxic-ischemic encephalopathy and perinatal strokes, neurocutaneous syndromes including Sturge-Weber syndrome, and Tuberous Sclerosis Complex, structural brain disorders, malformative syndromes, inborn errors of metabolism and as recently shown immunologic factors. Gene mutations have been recognized as a causative event of ISS. The first reports of gene mutation in ISS were linked to the Aristeless (ARX1) and the cyclin-depend Kinase-like (CDKL5) [79]. Genes frequently involved with ISS are the PAFAH1B1/LIS1, DCX, and TUBA1A855. Other genes implicated in the etiology of ISS are DKL5, STXBP1, KCNQ2, GRIN2A, MAG12, SPTAN, FOXG1, NSD1, WDR4 and RARS2 [60, 80–83]. There are multiple treatment options for ISS, which can be used together or individually under different situations. Hormonal, pharmacologic, ketogenic diet, and surgery are the eventual options for treatment. Adrenocorticotrophic hormone (ACTH) is widely used, with a wide range of dosage, but the most carried out is 2–3 IU/Kg/day. ACTH treatment is usually conducted for 3–4 weeks. Pharmacologic treatment is linked to the use of vigabatrin (50–125 mg/kg/day) alone or in association with other drugs. A ketogenic diet (ATKINS) is recommended by several authors, firstly by Kossofl, who report good results in more than 45% of the children treated with this diet [84]. Surgery is rarely used and restricted to cases of documented focal epileptogenesis and when pharmacologic treatment did not help [85].

#### Severe myoclonic epilepsy in infancy- Dravet syndrome

Since the initial description in 1978 [86] and subsequent reports of Dravet, the clinical features of this disorder are represented by severe myoclonic epilepsy with onset in infancy (SMEI), associated with multiple seizure types, last-during epileptic seizures with frequent episodes of status epilepticus, often triggered by fever [87, 88]. The incidence is reported as about 1 in 40,000 infants [89, 90]. In its classical clinical feature, patients affected by this disorder initially growth normally, and seizures begin around the age of 6–8 months trigger by fever, and presenting with either low and high temperature. Seizures are last –during and might have generalized or unilateral expression. Less commonly, seizures might also develop without fever. The EEG might be initially normal or present with diffuse or unilateral slowing after the episodes of prolonged course. There are five principal diagnostic criteria for classical DS: normal developmental before seizure onset; two or more febrile seizures complex before the age of 12 months; myoclonic, hemiclonic or generalized tonic-clonic seizures; two last-during seizures; and refractory seizures after the age of 2-years [88]. However, in patients with SMEI related to SCN1A gene mutations, some of the above-mentioned clinical signs might be absent, including the different age of onset, type of seizures, EEG pattern, and the non-involvement of intellective capacity [91–94]. For DS linked to SCN1A mutation, the term SCN1A-related epilepsy syndrome has been proposed. This syndrome might also show a wide clinical expression involving not only the brain but also cardiac, hearing, vision, movement issues, urinary, bowel, and endocrine functions [95]. Psychiatric disturbances and autistic behavioral have frequently been reported [95]. The EEG typically shows focal or multifocal spike-waves, sharp waves, and slow waves and spikes activity [88]. In approximately 70–80% of cases, DS is related to a genetic disorder, mostly carrying a de novo SCN1A mutation, and including truncating, missense, and splice- site mutations in 40%, 40%, and 20% of cases, respectively [96]. Other genes have been associated with DS-like phenotypes, including SCN2A, SCN8A, SCN9A, SCN1B, PCDH19, GABRA1, GABRAG2, STXBP1, HCN1, CHD2, and KCNA2 [95–100]. SCN1A mutations cause an inhibition of the GABAergic inhibitory interneurons, leading to excessive neuronal excitation. This model is referred to as the interneuron hypothesis and is the most accepted mechanism for DS [96–98]. The SCN1A genes encode nine mammalian voltage-gated sodium channel alfa subunits, and their mutation is one of the most common causes of epilepsy detected in 70% to 85% of patients with DS and 3% to 6% of patients with generalized epilepsy with febrile seizures plus (GEFS+) [91, 97–100]. SCN1A haploinsufficiency producing Nav1.1 dysfunction mainly affects GABAergic neurons, which according to the affected site, cortex, cerebellum, basal ganglia, or hypothalamus, are the cause of epileptic seizure, ataxia, crouching gait, thermal dysregulation, and sleep disturbances [96, 98, 101, 102]. The prognosis for DS is severe for both epileptic seizures and cognitive impairment, and the mortality rate is significant and half of the deaths are recorded as Sudden Unexpected Death (SUDEP) [103]. DS treatment is based on the use of appropriate drugs. Sodium channel blocking drugs are not advised in cases of DS due to the genetic defects affecting the sodium channels. In most cases, the drugs used in the treatment of DS produce little benefit. Pharmacological treatment attempts have been carried out with oxcarbamazepine, phenytoin, bromides, topiramate, levetiracetam, vigabatrin, stiripentol (STP), valproic acid, clobazam. [73] The effectiveness of STP was tested in 32 patients affected by DS, 15 of which had SCN1A mutations. The authors found that STP treatment was able to reduce the frequency of seizures in 72 ± 23% of the mutation group, compared to 50 ± 40% of the non-mutation group [104]. Triple treatment with valproate, topiramate, and STP has been effective in DS case report [105]. In a study of Myers et al. [106] performed on 41 patients with DS, treatment with STP displayed a long-term reduction of about 50% of cases both in patients with generalized than those with focal epileptic seizures. In general STP treatment in DS is effective and well-tolerated and markedly reduce the frequency of prolonged seizures [107]. New drugs have been proposed for the treatment of SD [108].

#### Myoclonic encephalopathies in nonprogressive disorder

The syndrome, also termed as “Myoclonic Status in Nonprogressive Encephalopathies (MSNE)” begins in early age, with an average age of 10 months [109, 110].

Three subgroups are recognized with a different presentation concerning the etiology, clinical aspect, EEG, and the evolution. In the first group, the etiology is genetic; the seizures are myoclonic or myoclonic absence-types. The EEG features consist of periodic theta-delta activity predominant in the central regions or might appear as brief runs of slow delta rhythm in the posterior regions. In the second group, the etiology is unknown with bilateral positive myoclonic jerks or relevant abnormal movements; the EEG shows diffuse slow background with status epilepticus or theta-delta rhythm prevalent in frontal regions. In the third group, the developmental delay is mild, and focal motor seizures involve mainly the face; the EEG displays generalized spike-wave paroxysms [111].

## Conclusions

The diagnosis of early-life seizures is complex, and includes conditions that can have a favorable course or dramatic effects. The onset of the first episode is cause of great concern for the parents and caregivers, and there is pressure for the pediatrician to express an immediate diagnosis. Correct diagnosis can help to produce appropriate treatment and accurate prognosis. The first problem is to differentiate epileptic seizures from abnormal non-epileptic movements. Video-registration from the parents might help with this distinction.

Early onset seizures are a clinical expression of various disorders with different etiologies and prognosis. The benign type of early-life seizures is associated with a normal physical and neurological examination, adequate developmental milestones, good eye contact, and prompt response to the archaic reflexes. In this regard, the absence of cerebral impairment is often a positive prognostic sign.

Epileptic seizures are usually accompanied by others abnormal clinical manifestations. A clinical examination might indicate the presence of anomalies on the skin, face, and body organs, indicating a neurocutaneous, a malformative syndrome, an inborn errors of metabolism or other neurologic disorders.

It is also relevant to note the frequency and complexity of seizure types which might be indicative of the severity of the disorders. Video-EEG registration are of notable importance as are the ophthalmologic examination and brain MRI. Recently, genetic analyses are revealing the gene mutations involved and will likely make relevant contributions to assignments of a correct diagnosis. In the group of epileptic encephalopathies, the electroclinical definition and diagnosis of each of the various disorders particularly in the precocious phases is relevant but not simple, because of the similarity of presentation and characteristic of seizures of the affected infants to shift from one type to another. Moreover, the electroclinical patterns reported in these patients might be influenced by several factors such as the causative event, the age of seizure onset, the time of EEG recording and the treatment already performed leading to inconclusive results on the type of epileptic disorders. All of the seizures belonging to the group of EE show resistance to pharmacological drugs, to hormonal treatments, and to ketogenic diet. Prognosis in EE patients is usually severe and mainly based on the causative event. Correct diagnosis, appropriate and precocious treatment, are the best way to improve the course of the disorders.

## Abbreviations

BFIS: Benign Familial Infantile Seizures
BFNE: Benign Familial Neonatal Epilepsy
DS: Dravet Syndrome
EIEE: Early Infantile Epileptic Encephalopathy
EIMFS: Epilepsy of Infancy with Migrating Focal Seizures
EME: Early Myoclonic Encephalopathy
FS: Febrile seizures
FSc: Febrile Seizures Complex
FSs: Febrile Seizures Simplex
GEFS+: Genetic Epilepsy with Febrile Seizures plus
ISS: Infantile Spams Syndrome
SRS: Situation-Related-Seizures
